# Chronic Kidney Disease in Lithuanian Children: Single Center Experience

**DOI:** 10.15388/Amed.2026.33.1.10

**Published:** 2026-07-22

**Authors:** Ugnė Rukšėnaitė, Karolis Ažukaitis, Rimantė Čerkauskienė, Dovilė Ruzgienė, Renata Vitkevič, Darija Litvinenko, Augustina Jankauskienė

**Affiliations:** 1 Vilnius University, Faculty of Medicine, Vilnius, Lithuania E-mail: ORCID ID; 2 Vilnius University, Faculty of Medicine, Lithuania E-mail: ORCID ID; 3 Vilnius University, Faculty of Medicine, Lithuania E-mail: ORCID ID; 4 Vilnius University, Faculty of Medicine, Lithuania E-mail: ORCID ID; 5 Vilnius University, Faculty of Medicine, Lithuania E-mail:; 6 Vilnius University, Faculty of Medicine, Vilnius, Lithuania E-mail: ORCID ID; 7 Vilnius University, Faculty of Medicine, Lithuania E-mail: ORCID ID

**Keywords:** Chronic kidney disease, CAKUT, children, CKD complications, Lėtinė inkstų liga, CAKUT, vaikai, LIL komplikacijos

## Abstract

**Background:**

*Chronic Kidney Disease* (CKD) in children is relatively rare but carries high burden due to increased morbidity and mortality rates. Unlike adults, where CKD is often secondary to other conditions, pediatric CKD is primarily caused by *Congenital Anomalies of the Kidney and Urinary Tract* (CAKUT). In Lithuania, data on pediatric CKD are limited, and center-specific patient characteristics and management practices remain underreported in real life settings. This study aimed to assess the current patterns of pediatric CKD in a major Lithuanian tertiary center and evaluate how current practice aligns with guideline recommendations.

**Material and Methods:**

A cross-sectional study of children (<18 years) with CKD stages 2–5 at Vilnius University Hospital Santaros Klinikos Pediatric Center was conducted. CKD stages were defined according to the *Kidney Disease: Improving Global Outcomes* (KDIGO) 2024 guidelines, and the patients were categorized into seven groups according to CKD etiology. Anthropometric measurements, complications, comorbidities, and ongoing treatments were analyzed.

**Results:**

A total of 65 children were included (mean age 11.1 years; male-to-female ratio 1.7:1). CAKUT was the most common etiology of CKD (61.5%), with renal hypoplasia being the predominant variant, followed by cystic kidney diseases (13.9%), glomerulopathies (9.2%), and other or unknown causes (9.2%). The mean estimated glomerular filtration rate (eGFR) was 60.5 mL/min/1.73 m^2^, and most children were in CKD stage 2 (66.2%). Differences in age, sex, eGFR, and the CKD stage across the etiology groups were not statistically significant (all *p* >0.05). Normal weight remained the predominant category at every CKD stage, with a prevalence of 60.0–66.7%. Nearly half of the patients had CKD-related complications, most commonly CKD-mineral and bone disorder, proteinuria, and arterial hypertension. Most children (76.9%) had received at least one treatment for CKD-related complications.

**Conclusions:**

Pediatric CKD in Lithuania remains predominantly caused by CAKUT, with most children diagnosed with CKD stage 2. Complications occur even in early stages, and the body mass index distribution does not vary significantly across the CKD stages. Management generally aligned with KDIGO guidelines, but gaps in treating proteinuria, anemia, and growth impairment remain.

## Abstract (it)

**Įvadas:**

Lėtinė inkstų liga (LIL) vaikams yra gana reta, tačiau susijusi su didele ligos našta dėl padidėjusio sergamumo ir mirtingumo. Skirtingai nei suaugusiesiems, kuriems LIL dažniausiai yra lėtinių ligų pasekmė, vaikai LIL dažniausiai serga dėl įgimtų inkstų ir šlapimo takų anomalijų (angl. *congenital anomalies of the kidney and urinary tract*, CAKUT). Lietuvoje duomenų apie vaikų LIL yra nedaug ir pacientų duomenys bei gydymo praktika vieno centro lygmeniu mažai ištirta. Šio tyrimo tikslas – įvertinti vaikų LIL duomenis dideliame Lietuvos tretinio lygio centre ir nustatyti, ar pacientų gydymas atitiko gairių rekomendacijas.

**Metodai ir medžiaga:**

Vilniaus universiteto ligoninės Santaros klinikų Pediatrijos centre buvo atliktas skerspjūvio tyrimas, įtraukiant vaikus (<18 metų), sergančius 2–5 stadijos LIL. LIL stadijos nustatytos pagal 2024 metų *Kidney Disease: Improving Global Outcomes* (KDIGO) gaires ir pacientai suskirstyti į septynias etiologines grupes pagal LIL priežastį. Analizuoti jų antropometriniai duomenys, komplikacijos, gretutinės ligos ir taikomas gydymas.

**Rezultatai:**

Tyrime dalyvavo 65 vaikai (vidutinis amžius 11,1 metų; berniukų ir mergaičių santykis 1,7:1). CAKUT buvo dažniausia LIL priežastis (61,5 %), vyraujantis CAKUT variantas ‒ inkstų hipoplazija. Kitos dažnos LIL etiologijos grupės: cistinės inkstų ligos (13,9 %), glomerulopatijos (9,2 %) ir kitos ar nežinomos priežastys (9,2 %). Vidutinis glomerulų filtracijos greitis (GFG) buvo 60,5 mL/min/1,73 m^2^, daugumai vaikų nustatyta 2 stadijos LIL (66,2 %). Amžiaus, lyties, GFG ir LIL stadijos skirtumai tarp etiologinių grupių nebuvo statistiškai reikšmingi (visi p > 0,05). Lyginant vaikų, sergančių skirtingomis LIL stadijomis, kūno svorį, 60,0–66,7 % jų buvo laikomi normalaus kūno svorio. Beveik pusei pacientų nustatytos su LIL susijusios komplikacijos, dažniausiai – su LIL susijęs mineralų ir kaulų apykaitos sutrikimas, proteinurija ir arterinė hipertenzija. Dauguma vaikų (76,9 %) vartojo bent vieną medikamentą LIL komplikacijoms gydyti.

**Išvados:**

Lietuvoje vaikų LIL priežastis vis dar dažniausiai išlieka CAKUT ir daugumai vaikų nustatyta 2 stadijos LIL. Komplikacijos pasireiškė net ankstyvose ligos stadijose. Kūno masės indekso pasiskirstymas statistiškai nesiskyrė tarp LIL stadijų. Gydymo praktika daugeliu atvejų atitiko KDIGO gairių rekomendacijas, tačiau proteinurijos, anemijos ir augimo sutrikimų gydymas išlieka ribotas.

## Introduction

*Chronic Kidney Disease* (CKD) is defined as abnormalities of the kidney structure or function, present for at least 3 months, with implications for health [1]. Although uncommon, CKD is an important cause of morbidity in children. Worldwide epidemiologic data remain limited, with previously reported prevalence ranging from 15 to 74.7 cases per million children [2], while European registries report a prevalence of 56–96 per million children [3].

The causes of CKD in children differ from those in adults. In adults, CKD is most often associated with other chronic conditions, including diabetes and *Arterial Hypertension* (AH), whereas, in children, it typically results from *Congenital Anomalies of the Kidney and Urinary Tract* (hereinafter abbreviated as CAKUT) [4,5]. Because pediatric CKD often develops early and affects kidneys during critical periods of growth, it can impair metabolic regulation and organ function [6,7]. Consequently, the affected children are at increased risk of CKD-related complications. Proteinuria, metabolic acidosis, hyperparathyroidism, AH, anemia, and hyperphosphatemia have all been associated with CKD progression [8].

In Lithuania, pediatric CKD was last analyzed in 2017 [9], providing the only national overview to date. Since then, significant developments have occurred, including the launch of the *European Rare Kidney Disease Registry* (ERKReg) [10] and the updated *Kidney Disease: Improving Global Outcomes* (KDIGO) guidelines [1], but real life data are still lacking. Our study presents nearly decade-later data from the country’s tertiary pediatric nephrology center, offering a detailed real-life perspective on etiology, complications, and treatment practices.

The aim of this study was to assess the current CKD patterns in children receiving care at a major Lithuanian tertiary center, while providing insight into the local burden of pediatric CKD and informing how the current practice aligns with guideline recommendations.

## Materials and Methods

### Study Design and Subjects

This single-center, cross-sectional study was conducted at Vilnius University Hospital Santaros Klinikos Pediatric Center on 2 January 2025.

Children aged <18 years who were regularly followed by the hospital’s pediatric nephrologists and had CKD stages 2–5 were eligible for inclusion. For each patient, the most recent available laboratory tests performed in 2024 were analyzed. Patients with a history of kidney transplantation were excluded. CKD was defined as an estimated glomerular filtration rate (eGFR) <90 mL/min/1.73 m^2^ persisting for at least three months. eGFR was calculated by using the updated Schwartz equation [11], and the CKD stages were determined according to the KDIGO 2024 Clinical Practice Guideline for the Evaluation and Management of Chronic Kidney Disease [1].

### CKD Etiology

The patients were categorized according to the CKD etiology into seven groups: glomerulopathies, tubulopathies, *Thrombotic Microangiopathies* (TMA), metabolic nephropathies, congenital anomalies of the kidney and urinary tract (CAKUT), familial cystic kidney diseases, and other or unknown causes. The ‘other or unknown causes’ category included patients with CKD of an unknown etiology as well as those patients who could not be classified into any of the other predefined groups. Within the CAKUT group, isolated CAKUT was defined as the presence of a single CAKUT, whereas complex CAKUT was defined as the presence of two or more congenital anomalies.

### Anthropometric Measurements

The *Body Mass Index* (BMI) was calculated as weight (kg) divided by height (m^2^). BMI was interpreted by using the *World Health Organization*’s (WHO) reference tables for children and adolescents aged 0–19 years [12,13]. According to the WHO z-score cut-offs, underweight was defined as < −2 *Standard Deviation* (SD), normal weight as −2 SD to +1 SD, overweight as > +1 SD, and obesity as > +2 SD. For some analyses, overweight and obesity were combined as ‘overweight/obese’.

### Comorbidities

Comorbidities were defined as any diagnoses other than the primary kidney disease documented in the patients’ medical records. The comorbidity status was recorded as present or absent. When applicable, comorbidities were further classified as congenital. Congenital comorbidities included isolated congenital anomalies, multiple congenital anomalies, and recognized genetic or syndromic conditions. The patients were categorized as having no congenital comorbidities, a single congenital anomaly, or multiple congenital anomalies.

### Complications and Treatment

Proteinuria was defined as persistent protein levels >0.3 g/L in spot urine, confirmed in more than one urine sample over time. AH was diagnosed according to the European Society of Hypertension 2016 pediatric guidelines [14]. The blood pressure was assessed through repeated measurements, including both office and home readings, following the recommendations provided in the guidelines. Hyperkalemia was defined as serum potassium >5.5 mmol/L, and metabolic acidosis was considered present when serum bicarbonate was <22 mmol/L [1]. Anemia was determined according to hemoglobin (Hb) thresholds for children with CKD recommended by the KDIGO 2012 guidelines [15]: children aged 0.5–5 years were considered anemic if Hb <110 g/L, those aged 5–12 years if Hb <115 g/L, those aged 12–15 years if Hb <120 g/L, and adolescents aged 15–18 years by using the adult thresholds (<130 g/L for males and <120 g/L for females). *CKD-Mineral and Bone Disorder* (CKD-MBD) was identified by abnormalities in serum calcium, phosphorus, parathyroid hormone or vitamin D levels based on the KDIGO 2017 guidelines [16]. A short stature was defined as a height below the 3^rd^ percentile for the age and sex based on the National growth reference data [17].

Medical treatments ongoing at the time of data collection included renoprotective therapy (RPT), antihypertensive therapy (AHT), potassium binders (PB), sodium bicarbonate (SB), erythropoietin (EPO), vitamin D, alfacalcidol, phosphate binders (PBs), other CKD-MBD-specific therapies, and growth hormone (GH).

### Ethics

All included patients were registered in the ERKReg system [10], and an informed consent had been obtained in advance. The study was conducted in accordance with the ethical standards of our institution.

### Statistical Analysis

The data were entered and organized by using *Microsoft Excel*. Statistical summaries were performed with the *R* software (version 4.3.3) by using the *Rcmdr* package (version 2.9.2). Descriptive statistics were used to summarize demographic and clinical characteristics. Continuous variables are presented as mean ± SD, and categorical variables are featured as frequencies and percentages. Differences in continuous variables (age, eGFR) between the etiology groups were assessed by using one-way analysis of variance. Associations between categorical variables (etiology group and sex; etiology group and CKD stage; BMI category and CKD stage) were evaluated by using the Pearson chi-square test. A *p*-value of <0.05 was considered statistically significant. The data were visualized by using graphs and tables. Graphs, including clustered column charts, a pie chart, and a stacked bar chart, were created by using *Microsoft Excel*. Tables were used to present detailed numerical data.

## Results

### General Characteristics

A total of 65 children with CKD were included in the study with a male-to-female ratio of 1.7:1 (see Table 1). More than half of the children had at least one comorbidity, including single or multiple congenital anomalies. Several syndromic conditions not primarily associated with a major kidney disease were identified, including three cases of Down syndrome and one case of Gilbert’s syndrome. The mean eGFR was 60.5 ml/min/1.73 m^2^. Two children (3.1%) received peritoneal dialysis.

**Table 1:** Baseline characteristics of the patients

Characteristic	Number of patients (n = 65) (%)
**Mean age (range), years**	11.05 (0‒17)
**Gender**	
- Boys	41 (63.08)
**Comorbidities**	
- Yes	44 (67.69)
- No	21 (32.31)
**Congenital comorbidities**	
- None	42 (64.62)
- Single anomaly	10 (15.38)
- Multiple anomalies	13 (20.00)
**CKD stage**	
- Stage 2	43 (66.15)
- Stage 3	10 (15.38)
- Stage 4	9 (13.85)
- Stage 5	3 (4.62)
Cause of CKD*	
- Glomerulopathies	6 (9.23)
- TMA	1 (1.54)
- Metabolic nephropathies	3 (4.62)
- CAKUT	40 (61.54)
- Isolated CAKUT	22 (33.85)
- Complex CAKUT	18 (27.69)
- Cystic kidney diseases	9 (13.85)
- Other or unknown causes	6 (9.23)
**BMI category**	
- Underweight	5 (7.69)
- Normal	42 (64.62)
- Overweight	12 (18.46)
- Obese	6 (9.23)

*Note*. The following abbreviations were used: SD – Standard deviation; CKD – Chronic kidney disease; CAKUT – Congenital anomalies of the kidney and urinary tract; TMA – Thrombotic microangiopathies; *Percentages do not sum to exactly 100% due to rounding.

### CKD Etiology

**Figure 1:**
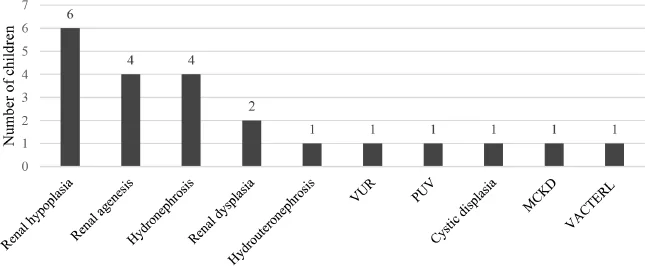
Distribution of isolated CAKUT variants in children *Note*. The following abbreviations were used: VUR – Vesicoureteral reflux; PUV – Posterior urethral valves; MCKD – Multicystic dysplastic kidney; VACTERL – Vertebral, anorectal, cardiac, tracheo-esophageal, renal, limb anomalies.

As shown in Table 1, more than a half of the children had CAKUT. Within this group, isolated CAKUT defects were identified in 55.0% of the children, while complex CAKUT anomalies were found in 45.0% of the patients surveyed. The types of isolated CAKUT anomalies in the cohort are presented in Figure 1. Among children with complex anomalies, vesicoureteral reflux (VUR) was observed in 9/18 children (50.0%), renal hypoplasia in 7/18 (38.9%), and hydronephrosis in 6/18 (33.3%).

The glomerulopathy group included children with IgA nephropathy associated with focal segmental glomerulosclerosis (FSGS) (2/6), one of whom also presented with rapidly progressive glomerulonephritis, nephritic syndrome with FSGS (1/6), FSGS (1/6), Alport syndrome (1/6), and an unspecified glomerulopathy (1/6). Metabolic nephropathies included nephrocalcinosis and Dent disease type 1. TMA was represented by a single case of typical hemolytic uremic syndrome. Cystic kidney diseases consisted mostly of polycystic kidney disease (4/9), juvenile nephronophthisis (2/9), and HNF1-beta nephropathy (2/9). One child had juvenile medullary cystic kidney disease associated with Joubert syndrome. Within the other or unknown causes etiology group, the etiology of CKD was unknown in all children, with one case described as nephropathy of an unknown origin. Three children had no identified comorbidities. Two children had CKD of an unknown etiology in the context of significant systemic and congenital conditions, as both had tetralogy of Fallot complicated by chronic heart failure. Notably, there were no cases of tubulopathies in this cohort.

Further etiological comparisons are shown in Table 2. Children with CAKUT were relatively young (10.6 years, range 0‒17), and predominantly male (67.5%). Patients with cystic kidney disease were the youngest (8.88 years, range 2‒17) and had the lowest mean eGFR (44.5 mL/min/1.73 m^2^), with 44.4% in CKD stages 4‒5. Glomerulopathy patients were the oldest (12.7 years, range 8‒17) with a mean eGFR of 69.8 mL/min/1.73 m^2^, mostly in stage 2 (83.3%). Although these clinical patterns differed across etiology groups, differences in age, sex distribution, eGFR, and CKD stage were not statistically significant (all *p* >0.05).

**Table 2:** Pediatric CKD Characteristics by etiology group

Etiology group	Mean age (range), years	Boys, n (%)	Mean eGFR ± SD (mL/min/1.73 m^2^)	CKD Stage 2, n (%)	CKD Stage 3, n (%)	CKD Stages 4–5, n (%)
Glomerulopathies (n=6)	12.67 (8‒17)	5 (83.33)	69.77**±**19.40	5 (83.33)	1 (16.67)	-
TMA (n=1)	17	0 (0.00)	18,60	-	-	1 (100)
Metabolic nephropathies (n=3)	15.67 (15‒16)	3 (100)	75.80**±**8.05	3 (100)	-	-
CAKUT (n=40)	10.60 (0‒17)	27 (67.50)	60.71**±**25.00	26 (65.00)	7 (17.5)	7 (17.5)
Cystic kidney diseases (n=9)	8.88 (2‒17)	4 (44.44)	44.47**±**29.07	4 (44.44)	1 (11.11)	4 (44.44)
Other or unknown causes (n=6)	12.33 (8‒15)	2 (33.33)	72.97**±**11.24	5 (83.33)	1 (16.67)	-

*Note*. The following abbreviations were used: SD – Standard deviation; eGFR – Estimated glomerular filtration rate; CKD – Chronic kidney disease; CAKUT – Congenital anomalies of the kidney and urinary tract; TMA – Thrombotic microangiopathies.

### Anthropometrics

**Figure 2:**
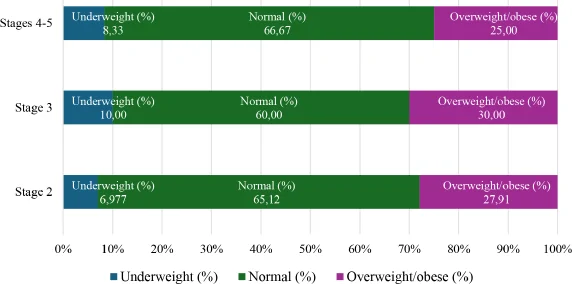
Distribution of BMI categories (Underweight, Normal, Overweight/Obese) in patients with CKD stages 2–5

BMI categories are summarized in Table 1, and their distribution across CKD stages is shown in Figure 2. Normal weight remained the predominant category at every CKD stage, with a prevalence of 60.0–66.7%. Overall, the distribution of BMI categories did not differ significantly across the CKD stages (*p* >0.5).

### CKD-Related Complications

**Figure 3:**
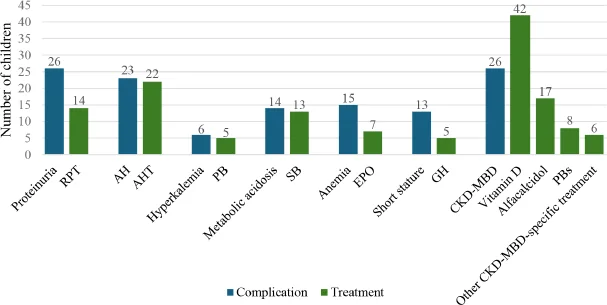
Distribution of complications and treatment in children with CKD *Note*. The following abbreviations were used: RPT – Renoprotective treatment; AH – Arterial hypertension; AHT – Antihypertensive therapy; PB – Potassium binders; SB – Sodium bicarbonate; EPO – Erythropoietin; GH – Growth hormone; CKD-MBD – Chronic kidney disease–mineral and bone disorder; PBs – Phosphate binders.

Nearly a half of the cohort (46%) experienced multiple CKD-related complications, whereas 32% had one complication, and 22% had no complications. As illustrated in Figure 3, the most frequent complications among the study population were CKD-MBD, proteinuria, and AH. A short stature occurred across the CKD stages as follows: 31% of the children in stage 2, 15% in stage 3, and 54% in stages 4–5. Anemia was observed in 7% of the children in stage 2, 20% in stage 3, and 73% in stages 4–5. Metabolic acidosis affected 14% of the children in stage 2, 7% in stage 3, and 79% in stages 4–5.

Overall, 76.9% of the patients received one or more medications. For several complications, including AH, hyperkalemia, and metabolic acidosis, the number of treated children closely approximated the number diagnosed. For proteinuria, approximately a half of the affected children received RPT. Similarly, anemia was treated in about a half of the children with EPO, and a short stature was treated in a minority of children with GH. Among children with CKD-MBD, most received vitamin D supplements (92.3%), whereas alfacalcidol (61.5%), PBs (30.8%), and other CKD-MBD-specific therapies (23.1%) were given less frequently. Eighteen children without CKD-MBD received active vitamin D therapy.

## Discussion

Pediatric CKD in Lithuania was previously analyzed in 2017 [9], and the present study provides updated data collected eight years later, while focusing on the experience of the largest tertiary pediatric nephrology centre in the country. Our findings confirm that CAKUT remains the most frequent cause of pediatric CKD in Lithuania, with a substantially higher proportion than previously reported, accounting for 61.5% of cases in the current cohort compared with 37.9% in 2017, which is a difference that may partly reflect changes in etiological classifications between the two studies. This pattern is similar to that reported by Harambat et al., in which, CAKUT accounted for 64.1% of cases among children with CKD stages 3–5 [18].

Although CAKUT is widely reported as the leading cause of pediatric CKD, the distribution of specific anomalies varies considerably across regions. Population-based studies from China, Iran, and Saudi Arabia have reported hydronephrosis as the most frequent anomaly (31.8–74.6%) [19–21], whereas, a German cohort identified renal hypoplasia/dysplasia in 65% out of 405 children [22]. Data from Ethiopia indicate posterior urethral valves as the most common anomaly (27.5%) [23]. In our cohort, renal hypoplasia was the most frequent CAKUT manifestation, observed in 32.5% of children with CAKUT, resembling the German experience, although the overall proportion was lower. These international differences may be influenced by regional variations in maternal exposure to teratogens, nutritional status, population age structures, access to prenatal diagnostics, and health-care infrastructure.

Most children in our cohort were diagnosed with CKD stage 2 (66.2%). In the 2017 Lithuanian study, 42.4% of the children had CKD stage 2 [9], although this lower proportion may partly reflect the inclusion of children with kidney transplants, who were more likely to be classified in advanced CKD stages. Compared with reports from low- and middle-income countries, a study from Nepal reported that 60% of children first presented at stage 5 [24], while a Syrian cohort found that 48% of patients initially presenting at stage 4 [8]. Whereas, our findings suggest a more timely recognition and on-time referral. This pattern aligns with reports from European centers, where routine prenatal and neonatal ultrasound screening combined with close collaboration between nephrology and urology specialists facilitates an earlier identification and follow-up of children with CAKUT [25,26].

BMI is widely used as a primary screening measure of the nutritional status in children with CKD [27]. According to data from the Institute of Hygiene, the prevalence of overweight and obesity in the general Lithuanian pediatric population in 2022–2023 was 19.9% [28], which has been attributed to increasing sedentary behavior and unhealthy dietary habits among children [29,30]. In our cohort, the proportion of children classified as overweight or obese was only slightly higher (27.7%) than that observed in the general pediatric population. Most children had a normal BMI, and the BMI distribution did not differ significantly across the CKD stages. However, previous studies have shown that BMI does not distinguish between the fat mass and the lean mass, and that children with CKD often have increased fat-to-muscle ratios and a reduced lean mass despite the normal BMI values [31–33]. These findings suggest that, although BMI appeared largely preserved in our patients, more detailed assessment of body composition may be clinically relevant, particularly in advanced CKD stages.

Despite a predominance of mild-to-moderate CKD in our cohort, the burden of CKD-related morbidity was substantial. Nearly 80% of the children had at least one complication, and almost a half experienced multiple complications, thereby demonstrating that clinically relevant systemic involvement begins early in the disease course. Previous pediatric CKD cohorts have reported a broad spectrum of complications similar to those observed in our study [8,9,34]. The prevalence of AH, anemia, and a short stature in our cohort was comparable to findings from the multicenter cross-sectional study by Hsin-Hsu Chou et al., which included 757 children across all CKD stages [34]. Consistent with reports from the Lithuanian national pediatric CKD study and the cohort described by Harambat et al., the prevalence of AH, metabolic acidosis, and hyperkalemia increased with an advancing CKD stage, which reflects greater metabolic instability in later disease stages [9,18]. CKD-MBD was one of the most frequent systemic complications in our cohort, which falls in line with previous pediatric studies highlighting its central role in CKD [35,36]. Notably, CKD-MBD was observed even in children with a relatively preserved kidney function (eGFR >60 mL/min/1.73 m^2^), indicating that CKD-related morbidity may begin early in the disease course [36]. Proteinuria was the second most common complication in our population. This is consistent with data from a cohort of 90 children with CKD, in which, only 33% had normal or mildly increased proteinuria [37], and with a larger study showing an increase in proteinuria prevalence from 5.8% in early CKD to 40% in advanced stages (*p* <0.0001) [36]. A short stature affected approximately one-fifth of the cohort, despite the majority of the children being in early stages of CKD. This finding may reflect the influence of additional factors beyond CKD severity, as some children were born prematurely or had underlying conditions including Down syndrome [38,39]. As expected, the burden of complications increased progressively with an advancing disease severity. Congenital heart disease and obesity were observed in our cohort and are known to adversely influence CKD outcomes [40,41]. However, their precise impact could not be quantified due to the cross-sectional study design. In addition, a small subgroup of patients had syndromic conditions associated with renal involvement, further contributing to clinical complexity.

Our results highlight important aspects of real-world therapeutic practice in pediatric CKD. Overall, the management of several complications in our cohort largely reflected KDIGO guideline recommendations [1,15,16]. Treatment of AH, hyperkalemia, and metabolic acidosis closely matched the number of diagnosed cases, indicating generally adequate recognition and management of these complications. However, important gaps remained for other key complications. Only about a half of the children with proteinuria received RPT, despite proteinuria being a common CKD complication [36]. In selected children, combined RPT with angiotensin-converting enzyme inhibitors and angiotensin receptor blockers was used at the highest approved doses, and this strategy has been reported to be safe and potentially effective in reducing proteinuria and stabilizing the renal function [42]. Anemia in pediatric CKD has been associated with a reduced quality of life and an increased morbidity and mortality [43]. In our cohort, fewer than a half of children with anemia were treated with EPO, thereby likely reflecting a conservative approach in cases of mild anemia, with iron, folate, or vitamin B12 supplementation provided whenever appropriate [15]. Treatment of growth impairment was limited, with only a minority of the children with a short stature receiving GH in our cohort. According to the results from a North American cross-sectional survey, this is often due to the family refusal and variability in the clinical practice, despite consensus guidelines and evidence supporting its benefits [44]. Serum bicarbonate was demonstrated to be an important factor in CKD progression, as low levels were associated with an increased risk, particularly in children with glomerular disease, and the resolution of low bicarbonate was linked to a lower risk of CKD progression [45]. Management of CKD-MBD in our cohort was characterized by the widespread use of vitamin D supplementation, whereas other treatments were prescribed less frequently. Notably, a subgroup of children without formal biochemical criteria for CKD-MBD received active vitamin D therapy. This practice may reflect the high prevalence of vitamin D deficiency in the general pediatric population in Lithuania [46] and a preventive strategy aimed at delaying the development of secondary hyperparathyroidism in children with CKD stages 2–3 [47]. An adult study demonstrated potential renoprotective and antiproteinuric effects of vitamin D supplementation [48], supporting the use of active vitamin D analogues, including alfacalcidol, in children with reduced eGFR (<60 mL/min/1.73 m^2^) to help protect kidney function in early CKD stages. Our findings emphasize that pediatric CKD is associated with a high burden of early complications, even when the kidney function is relatively preserved. Early recognition of complications and systematic implementation of guideline-based interventions may help reduce long-term morbidity and slow disease progression in children with CKD.

## Strengths and Limitations

A major strength of this study is that it provides contemporary, real-world data from the largest tertiary pediatric nephrology center in Lithuania, offering a comprehensive overview of pediatric CKD. This study enables consistent etiological subgrouping and evaluates complications, comorbidities, BMI, and adherence to KDIGO guidelines [1], thereby providing a detailed insight into the current clinical practice.

However, several limitations should be acknowledged. The cross-sectional design precludes assessment of the disease progression and long-term outcomes. The relatively small cohort size limits statistical power to detect differences between etiological subgroups. In addition, the exclusion of transplant recipients may have resulted in an underestimation of the true national burden of CKD.

## Conclusions

This study highlights that CAKUT remains the leading cause of pediatric CKD, with most children diagnosed with CKD stage 2. Notably, complications can occur even in the early stages of the disease. To assess the potential impact of CKD on the children’s physical development, BMI was evaluated, as growth is a critical aspect of childhood, but no significant differences were observed between the CKD stages. Despite a predominance of mild-to-moderate CKD, nearly a half of the children experienced multiple complications, with CKD-MBD, proteinuria, and AH among the most frequent issues. Management of the blood pressure, electrolytes, and metabolic acidosis generally aligned with the KDIGO guidelines, but important gaps were observed in the treatment of proteinuria, anemia, and growth impairment, highlighting opportunities for more systematic, evidence-based interventions.

## List of Abbreviations

AH – Arterial hypertension

AHT – Antihypertensive therapy

BMI – Body mass index

CAKUT – Congenital anomalies of the kidney and urinary tract

CKD – Chronic kidney disease

CKD-MBD – Chronic kidney disease–mineral and bone disorder

eGFR – Estimated glomerular filtration rate

EPO – Erythropoietin

ERKNet – European Rare Kidney Diseases Reference Network

ERKReg – European Rare Kidney Disease Registry

FSGS ‒ Focal Segmental Glomerulosclerosis

GH – Growth hormone

Hb – Hemoglobin

KDIGO – Kidney Disease: Improving Global Outcomes

MCKD – Multicystic dysplastic kidney

PB – Potassium binders

PBs – Phosphate binders

PUV – Posterior urethral valves

RPT – Renoprotective therapy

SB – Sodium bicarbonate

SD – Standard deviation

TMA – Thrombotic microangiopathies

VACTERL – Vertebral, anorectal, cardiac, tracheo-esophageal, renal, limb anomalies

VUR – Vesicoureteral reflux

WHO – World Health Organization
